# PELICAN-IPC 2015-016/Oncodistinct-003: A Prospective, Multicenter, Open-Label, Randomized, Non-Comparative, Phase II Study of Pembrolizumab in Combination With Neo Adjuvant EC-Paclitaxel Regimen in HER2-Negative Inflammatory Breast Cancer

**DOI:** 10.3389/fonc.2020.575978

**Published:** 2020-11-25

**Authors:** Alexandre Bertucci, François Bertucci, Christophe Zemmour, Florence Lerebours, Jean-Yves Pierga, Christelle Levy, Florence Dalenc, Julien Grenier, Thierry Petit, Marguerite Berline, Anthony Gonçalves

**Affiliations:** ^1^ Département d’Oncologie Médicale, Institut Paoli-Calmettes, Centre de Recherche en Cancérologie de Marseille (CRCM), INSERM UMR1068, CNRS UMR7258, Aix-Marseille Université, Marseille, France; ^2^ Laboratoire d’Oncologie Prédictive, CRCM, Institut Paoli-Calmettes, INSERM UMR1068, CNRS UMR7258, Aix-Marseille Université, Marseille, France; ^3^ Aix-Marseille Université, Faculté de Médecine, Marseille, France; ^4^ Département d’Oncologie Médicale, Institut Curie, Saint-Cloud, France; ^5^ Département d’Oncologie Médicale, Institut Curie, Université de Paris, Paris, France; ^6^ Breast Cancer Unit, François Baclesse Cancer Centre, Caen, France; ^7^ Département d’Oncologie Médicale, Institut Claudius Regaud, Institut Universitaire du Cancer, Oncopole Toulouse, France; ^8^ Département d’Oncologie Médicale, Institut Sainte Catherine, Avignon, France; ^9^ Département d’Oncologie Médicale, Centre Paul-Strauss, Strasbourg, France; ^10^ Oncodistinct Network, Bruxelles, Belgium

**Keywords:** immune checkpoint inhibitor, inflammatory breast cancer, neoadjuvant therapy, PDL1, pembrolizumab

## Abstract

Inflammatory breast cancer (IBC) is a highly aggressive entity with a poor outcome and relative resistance to treatment. Despite progresses achieved during the last decades, the survival remains significantly lower than non-IBC. Recent clinical trials assessing PD-1/PD-L1 inhibitors showed promising results in non-IBC. Pembrolizumab, an anti-PD-1 monoclonal antibody, revolutionized the treatment of different cancers. Several recent studies suggested a potential interest of targeting the immune system in IBC by revealing a more frequent PD-L1 expression and an enriched immune microenvironment when compared with non-IBC. Here, we describe the rationale and design of PELICAN-IPC 2015-016/Oncodistinct-003 trial, an open-label, randomized, non-comparative, phase II study assessing efficacy, and safety of pembrolizumab in combination with anthracycline-containing neoadjuvant chemotherapy in HER2-negative IBC. The trial is ongoing. The primary endpoint is the pCR rate (ypT0/Tis, ypN0) in overall population and the co-primary endpoint is safety profile during a run-in phase. Key secondary objectives include tolerability, invasive disease-free, event-free and overall survivals, as well as collection of tumor and blood samples for translational research.

**Clinical Trial Registration:**

https://clinicaltrials.gov/ (NCT03515798).

## Introduction

Inflammatory breast cancer (IBC) is an uncommon (less than 5% of all BC) and very aggressive form of locally advanced BC. IBC has a clinical definition, which includes a rapidly (less than 6 months) enlarging, erythematous (which has to occupy at least one-third of the breast) and edematous breast (known as “peau d’orange”), which often presents without any underlying breast mass (1–3). Women with IBC are typically diagnosed at a younger age than patients with non-IBC (4, 5). IBCs are more frequently ductal than non-IBCs, with more frequent high grade, axillary lymph node involvement and metastases (more than 30%) at diagnosis (6, 7).

Biologically also, IBCs differs from non-IBCs, with more frequent hormone receptor (HR)-negativity and HER2-positivity (˜40% *versus* 15% in non-IBC) (8, 9), and a more angiogenic phenotype (10). IBCs display higher vascularity and an increased microvessel density (11, 12), and frequently include the presence of dermal lymphovascular emboli (13, 14). During the last two decades, IBC clinical tumor samples have been profiled using high-throughput molecular profiling technologies, mainly based on transcriptome analysis, in order to better delineate the molecular biology of disease (15). In 2013, the World IBC Consortium identified a robust 79-gene expression signature discriminating IBCs *versus* non-IBCs samples independently form the molecular subtypes (16). This signature notably suggested that alterations in TGF-β and immune response pathways are involved in the biology of IBC. Therefore, a particular tumor immune microenvironment is likely to participate into the unique biological patterns associated with IBC. Such importance of the tumor stroma has then been underlined by other research groups (17).

Significant therapeutic progresses were achieved during the past 50 years using a multidisciplinary approach, including neoadjuvant chemotherapy (NACT), followed by surgery and radiation therapy, and adjuvant anti-HER2 treatment and/or endocrine therapy –when indicated. However, the survival of IBC patients, when matched stage for stage, remains inferior to that of non-IBC patients. Research efforts are ongoing for many years to improve the treatment of disease. Due to the scarcity of the disease, its rapid progression and its unfavorable outcome, IBC-specific clinical trials have been rare. When they are not excluded, IBC patients are included in non-specific studies, being considered as locally advanced BC. Here, we present the rationale and the design of PELICAN-IPC 2015-016/Oncodistinct-003 trial, an open-label, multicentric, randomized, non-comparative, phase II study evaluating efficacy and safety of pembrolizumab in combination with neoadjuvant chemotherapy in HER2-negative IBC.

### Neoadjuvant Chemotherapy in IBC

Historically, the long-term survival was dramatically low (<5%) when patients were treated with loco-regional treatment only, suggesting the strong metastatic potential of IBC. Incorporation of multi-agent NACT in the therapeutic strategy significantly improved the prognosis, and achievement of pathological complete response after chemotherapy was identified as a favorable prognostic factor.

Advances in IBC have been made paralleling locally advanced non-IBC such as multi-agent NACT including anthracycline-based regimen with addition of taxanes, and more recently with incorporation of anti-HER2 targeted therapies (trastuzumab, pertuzumab, neratinib, trastuzumab-emtansine) in HER2 amplified disease (18–22). Three IBC-specific trials evaluated addition of bevacizumab in HER2-positive (23) and HER2-negative (24) IBC in the neoadjuvant and adjuvant setting, and panitumumab (an anti-EGFR monoclonal antibody) in HER2-negative IBC in the neoadjuvant setting (25). Results were promising with bevacizumab in HER2-positive and with panitumumab in triple-negative (TN) IBC, but these drugs are not recommended in routine. To our knowledge there is no ongoing IBC-specific study further evaluating these agents. Importantly, these trials showed the feasibility of IBC-dedicated clinical trials, with more than 50 patients enrolled per year in the two French multicentric trials (23, 24). However, and despite the benefit of NACT, the results are insufficient, with a 5-year survival remaining between 30% and 50%. Thus, it remains crucial to improve the results by optimizing neoadjuvant systemic regimen.

### Immune Microenvironment in IBC

Escape from immune destruction is an important way set up by cancers to promote cell transformation and favor tumor growth, which has been known for decades in various tumor models. In BC, this process was more recently enlightened. Thus, various features associated with immune response have a significant predictive impact on therapeutic efficacy and survival. In BC and also IBC, tumor infiltrating lymphocytes (TIL) (26–28) and immune gene expression signatures have shown a prognostic impact, in particular for ER-negative and/or high proliferating tumors (29–32). Interestingly, studies on small BC series have also indicated that some NACT regimens, such as anthracyclines-taxanes combinations, could favor the attraction of lymphocytes to the tumor bed (33, 34). The Programmed cell death 1 (PD-1) receptor-ligand interaction is a major inhibitor pathway hijacked by tumors to suppress immune control (35–41). Under physiological conditions, when PD-1, which is expressed on the cell surface of activated T-cells, is engaged by its ligands, Programmed death-ligand 1 and 2 (PD-L1 and/or PD-L2), it mitigates lymphocyte activation and promotes T-regulatory cell development and function, allowing to terminate the immune response. PD-L1 and PD-L2 are either constitutively expressed or induced in various tissues, including different neoplastic diseases. PD-L2 regulates T-cell activation in lymphoid tissues, whereas PD-L1 serves to limit unneeded T-cell function in peripheral organs and tissues. Several studies have examined PD-L1 expression in BC at ARN and protein levels, using different scoring systems: various expression rates have been reported ranging from less than 2% to 55%, with discordant prognostic impact (42–49). Our group retrospectively analyzed *PD-L1* mRNA expression in 45 BC cell lines and 5,454 clinical BC. Compared to normal tissue, we found PD-L1 expression as increased in 20% of clinical samples, and in almost 40% of basal-like subtypes. Expression of PD-L1 was associated with biological evidences of major cytotoxic immune response, such as TCR-related gene expression, indicative of a high T-cell infiltration. PD-L1 overexpression was not associated with survival in the overall population, but with better metastasis-free survival (MFS) and overall survival (OS) in basal-like tumors, independently from the clinico-pathological features. The pCR rate after NACT was higher in case of increased PD-L1 expression (50% *versus* 21%) (48).

Few studies have been specifically dedicated to IBC. In the World IBC Consortium series including 87 informative IBC samples (50), we identified and validated a robust 107-gene signature associated with pCR and strongly enriched for genes involved in both adaptative and innate immunity. In a cohort of 306 BC samples (51), including 112 IBC samples, PD-L1 was overexpressed in 38% of IBC samples compared to normal breast tissue. Such overexpression correlated with aggressive molecular subtypes (TNBC or basal-like and HER2-positive subtypes) and with a higher pCR rate to NACT as well as biological signs of antitumor T-cell cytotoxic response. There was no correlation with MFS and specific OS. Microenvironment of “PD-L1-high” IBC samples was in favor of a strong local cytotoxic immune response, with higher expression of T-cell-specific and CD8+ T-cell-specific gene signatures, and higher expression of T-cell receptor-related genes. In addition, these tumors displayed features of T-cell activation. However, some T-cells infiltrating the tumor had a phenotype of exhausted T-cells. Similar observations were reported at the protein level (52). In a recent study including 143 patients with IBC and 142 control subtype-matched patients with non-IBC, PD-L1 IHC expression on immune cells (SP142 antibody) was more frequent in IBC (42.9%) than in non-IBC (23.7%), and correlated with higher pCR rate and stromal TIL infiltration (53). This later was associated with improved overall survival in a multivariate model. Finally, recent next-generation sequencing studies have shown that IBC samples display higher tumor mutational burden (TMB) than non-IBC samples, independently from the molecular subtypes and tumor stage (54, 55). Such increased TMB in IBC might lead to increased tumor antigen-based attraction of cytotoxic T-cells and better sensitivity to immune checkpoint inhibitors.

### Pembrolizumab and Other Anti-PD1/PD-L1 Agents in BC

Pembrolizumab, a humanized immunoglobulin (IgG4) monoclonal antibody (mAb), binds PD-1 with a high specificity, blocks the interaction with PD-L1 and PD-L2, and reactivates inhibited T-cells, which is expected to increase the antitumor immune response. This drug and other immune checkpoint inhibitors (ICI) targeting the PD-1/PD-L1 axis showed evidences of antitumor activity in several cancers, with a favorable toxicity profile compared to conventional chemotherapy. They are already registered in various indications, especially in the management of non-small cell lung cancer, melanoma, renal carcinoma, and classical Hodgkin lymphoma.

When administered as single agent in advanced BC, pembrolizumab, and other ICI such as atezolizumab or avelumab generated moderate but detectable antitumor activity, with objective response rate ranging between 3 to 18% (56). Of note, efficacy was higher in patients with TNBC, minimal pre-treatment exposure, and PD-L1- and/or TILs-positive tumors. Yet, in the KEYNOTE119 randomized phase III study involving pre-treated advanced TNBC, pembrolizumab was not better than chemotherapy at physician’s choice (57).

There is also a solid rationale to combine anti-PD1/PD-L1 agents with chemotherapy in BC, which may have significant immunomodulatory effects, and may in turn increase the antitumor activity of PD-1 pathway inhibition (58, 59). Indeed, even though cytotoxic drugs have historically been considered as immunosuppressive, they can also have pro-immune properties (60–67) by i) depleting immuno-suppressive cells, including regulatory T-cells and myeloid-derived suppressor cells, which stimulate a quiescent anti-tumor immune response, ii) inducing an immunogenic cell death, iii) improving presentation of tumor antigens by upregulating their expression or that of the major histocompatibility complex (MHC) class I molecules, iv) up-regulating co-stimulatory molecules (B7-1) or down-regulating co-inhibitory molecules (PD-L1 or B7-H4) expressed on tumor or immune cells, thus boosting the activity of T-cell effectors, and vi) enhancing tumor cells sensitivity to T-cell–mediated lysis through fas-, perforin-, and granzyme B–dependent mechanisms.

Recent results from clinical studies in TNBC have confirmed the potential for combination of chemotherapy and ICI. First, in the IMpassion130 phase III randomized study, first-line atezolizumab plus nab-paclitaxel improved progression-free survival over nab-paclitaxel alone in advanced TNBC. Benefit was restricted to patients with PD-L1-positive tumors, in which a strong numerical advantage in OS was suggested, leading to approval by both FDA and EMA (68). Very recently, first results of the KEYNOTE-355 phase III randomized trial (69), comparing several chemotherapy regimens (nab-paclitaxel, paclitaxel or carboplatine plus gemcitabine) plus placebo *versus* chemotherapy plus pembrolizumab in the same setting, have confirmed IMPassion130 results in terms of progression-free survival for patients with PD-L1 combined Positive Score (CPS) 10. However, statistical significance was not achieved in PD-L1 CPS 1 patients and OS data are still immature. In addition, IMpassion 131 failed to demonstrate any advantage for atezolizumab in combination with paclitaxel over paclitaxel alone (70). IMpassion 132, which evaluates atezolizumab with capecitabine or carboplatin-gemcitabine is still ongoing (71). Second, a significant improvement in pCR rate was recently reported when pembrolizumab was added to NACT (carboplatin/paclitaxel followed by AC) in non-metastatic TNBC, while preliminary analysis suggested a possible and promising advantage in event-free survival (72, 73). A similar improvement in pCR was recently demonstrated with atezolizumab when combined with anthracyclines/taxanes but carboplatin-free NACT in IMpassion 031 trial (74). Yet, results from other studies evaluating anti-PD-L1 antibodies such as durvalumab, another anti-PD-L1 antibody in combination with anthracyclines/taxanes (75), or atezolizumab in combination with anthracyclines-free regimen (NeoTripp trial: NCT002620280) NACT in TNBC failed to significantly improve pCR rates (76). Thus, the role of ICI in NACT of early BC remains to be defined. Of note, in both advanced and early settings, no new signal of toxicity was detected, and tolerance was similar to what expected with ICI in other tumor types.

## Methods of PELICAN-IPC 2015-016/ONCODISTINCT-003 Study

The currently insufficient results of NACT in IBC, the relatively peculiar immune microenvironment of IBC when compared to non-IBC, and efficacy of pembrolizumab in BC led us to launch the PELICAN trial.

### Study Design and Participants

PELICAN-IPC 2015-016/Oncodistinct-003 is a prospective, multicenter, open-label, randomized, non-comparative, phase II study evaluating pembrolizumab in combination with NACT in HER2-negative IBC. The trial was registered in ClinicalTrials.gov database (NCT03515798). The study, promoted by Institut Paoli-Calmettes (Marseille, France) is being conducted in up to 21 centers (13 in France, 8 in Belgium), 10 of them being activated on January 2020. Patients are eligible to the study, if they have a previously untreated, histologically-confirmed diagnosis of HER2-negative IBC as defined according to 8^th^ American Joint Committee on Cancer (AJCC) classification: breast erythema, edema and/or *peau d’orange*, occupying at least 1/3 of the breast, with or without underlying palpable mass, duration of history of no more than 6 months. The main other inclusion and exclusion criteria are listed in **Table 1**. In PELICAN trial, all HER2-negative IBC patients are eligible, resulting in a mixed population of triple-negative and hormone receptor-positive/HER2-negative tumors. While we acknowledge that this is a significant limitation of the study, this is justified by the rarity of the disease and the anticipated difficulties of recruitment if restricted to a single IBC subtype. In addition, previous studies in the field, which provided the basis to our statistical hypothesis, were performed in a similar setting. Moreover, the design includes stratification on hormone receptor status and will allow a specific analysis in triple negative subtype, the most likely to benefit according to recent results (see Statistics section).

**Table 1:** Eligibility criteria of the PELICAN-IPC 2015-016/Oncodistinct-003 trial.

Inclusion criteria - HER2 negative tumors by immunohistochemistry (IHC 0 or 1+) or fluorescent/chromogenic *in situ* hybridization (FISH- or CISH-) - Hormone receptors status known - Previously untreated, histologically confirmed diagnosis of breast cancer and confirmed inflammatory breast cancer - No metastases - No organ dysfunction, especially adequate cardiac, kidney, liver and hematologic function - At least 18 years - Performance status (ECOG) 0 or 1. ECOG 2 may be considered if good rationale provided and discussed - A female participant if she is not pregnant, not breastfeeding, if she is a woman of childbearing potential (WOCBP), who agrees to the follow contraceptive guidance during the treatment period and for at least 12 months after the last dose of cyclophosphamide and 4 months after the last dose of pembrolizumab, whichever come last. Abstinence is acceptable - A male participant must agree to use a contraception during the treatment period and for at least 6 months after the last dose of study treatment and refrain from donating sperm during this period
Exclusion criteria - Bilateral breast cancer - Prior allogeneic stem cell or solid organ transplantation - WOCBP who has a positive serum pregnancy test within 72 h prior to randomization - Current participation in or recent participation in a study of an investigational agent or use of an investigational device within 4 weeks prior to the first dose of study treatment - Active CNS disease or carcinomatous meningitis - Diagnosis of immunodeficiency or is receiving systemic steroid therapy (in dosing exceeding 10 mg daily of prednisone equivalent) or any other form of immunosuppressive therapy within 7 days prior to the first dose of study drug, - Known history of active bacillus tuberculosis - Severe hypersensitivity (grade 3) to pembrolizumab and/or any of its excipients - Known additional malignancy that is progressing or has required active treatment within the past 3 years. Note: participants with basal cell carcinoma of the skin, squamous cell carcinoma of the skin, or carcinoma in situ (e.g., breast carcinoma, cervical cancer *in situ*) that have undergone potentially curative therapy are not excluded. - Active autoimmune disease that has required systemic treatment in the past 2 years - Active infection requiring systemic therapy or history of Human Immunodeficiency Virus, Hepatitis B or C - Delivery of a live vaccine within 30 days prior to the first dose of study drug - Known psychiatric or substance abuse disorders that would interfere with cooperation with the requirements of the trial - Prior therapy with an anti-PD-1, anti-PD-L1, or anti PD L2 agent or CTLA-4

### Study Procedures and Treatment

Patients are to be randomly assigned within 28 days from initiation of screening with a 2/1 ratio between NACT without (arm A) or with (arm B) pembrolizumab. The randomization procedure is assessed with block and is stratified by centers and hormone receptor status (positive HR is defined as tumor cell staining by immunohistochemistry ≥10% for ER and/or PR). To increase the randomness of the assignments, the permuted-block randomization schedule is generated within varying block sizes. A minimum and maximum number of patients of each phenotype (TN/non-TN) are respected in order to keep the adequate power.

**Figure 1:**
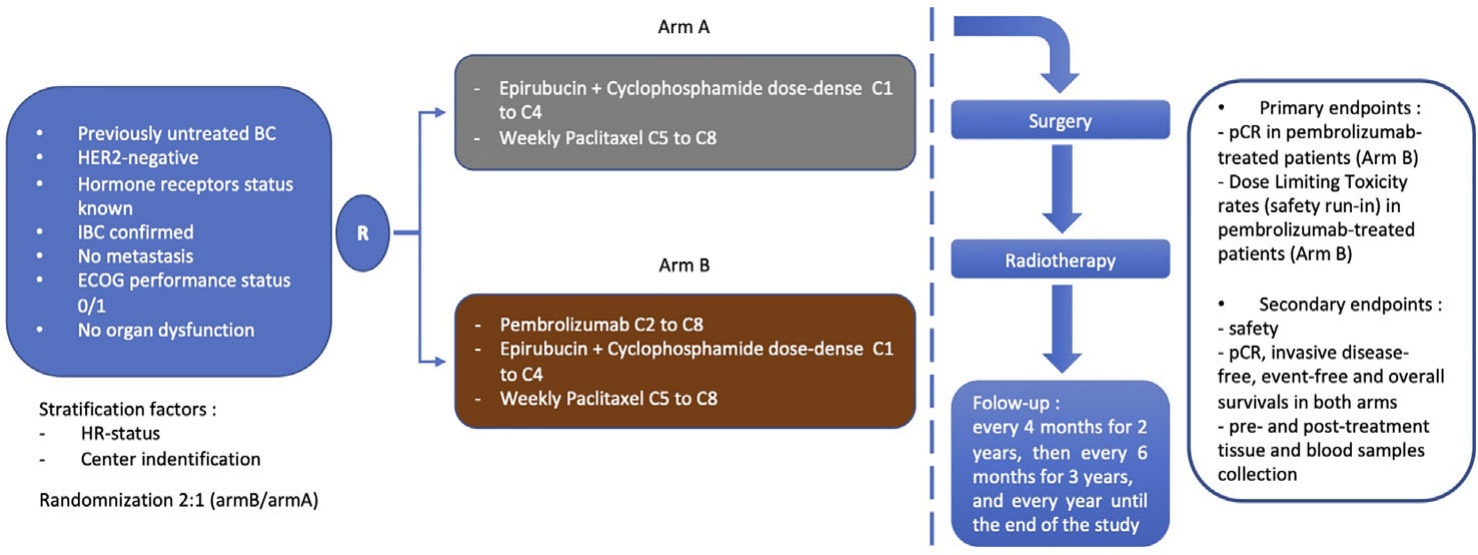
Design of PELICAN-IPC 2015-016/Oncodistinct-003 trial. BC, breast cancer; IBC, inflammatory breast cancer; HR, hormone receptor; pCR, pathological complete response.

In the experimental arm, pembrolizumab (intravenous administration at a dose of 200 mg every 3 weeks starting on cycle 2) is combined with conventional anthracycline/taxane-based NACT. Since anthracyclines have been shown to strongly induce immunogenic cell death, IFN gamma production and dendritic and T-cell tumor infiltration in mouse models (60, 61, 64, 66, 67), pembrolizumab is started on cycle 2, assuming that it should maximize potential sequential synergism. In addition, differing pembrolizumab initiation on cycle 2 should help evaluating the safety of the combination, by identifying patients with specific chemotherapy-induced toxicities. In the initial version of the protocol, the anthracyclines part of NACT was different according to the HR status: non-TN IBC (HR-positive and HER2-negative) patients were to receive 3-weekly 5-fluorouracil 500 mg/m², epirubicin 100 mg/m², cyclophosphamide 500 mg/m² (FEC100) from C1 to C4, whereas TN IBC (HR-negative and HER2-negative) were to receive epirubicin 90 mg/m² and cyclophosphamide 600 mg/m² every 2 weeks with G-CSF support, i.e., a dose dense (DD-EC) regimen from C1 to C4. A subsequent amendment homogenized the anthracycline-based schedule and all patients are now to receive DD-EC, whatever the HR status. Following anthracyclines sequence, all patients receive weekly paclitaxel for 12 injections (from C5 to C8). Details of design are shown in **Figure 1**.

Mastectomy with axillary lymph node dissection is to be performed within 4–6 weeks after the last chemotherapy administration. Pathological analysis is assessed by the local pathologist of each center and by a centralized reviewer. Radiotherapy starts 3 to 6 weeks after the surgery. Dose and frequency are left to investigator’s discretion and according to the site’s standard practice. Adjuvant endocrine therapy (if HR-positive disease) and/or capecitabine (if residual disease in TN IBC and no DPD enzyme deficit identified by plasma uracil dosage) is to be given after radiotherapy completion according to the site’s standard practice. After local treatment is completed, patients are followed every 4 months (+/- 28 days) for 2 years, then every 6 months for 3 years, and every year until the end of the study.

### Outcomes

The primary endpoint is a central evaluation of pCR rate (as defined as ypT0/Tis, ypN0 following 8^th^ AJCC classification) of the resected breast specimen and all sampled ipsilateral lymph nodes following NACT with/without pembrolizumab. A co-primary endpoint of safety is also included to determine if combining pembrolizumab and DD-EC exposes to significant toxicity. Thus, a run-in safety phase is to be conducted in order to stop the trial in case of unacceptable toxicity, as defined by the incidence of dose limiting toxicities (DLTs).

The secondary endpoints include the safety profile and tolerability of the combination, pCR rate by local assessment, invasive disease-free, event-free, and overall survivals (iDFS, EFS, and OS) in each arm. Since identification of predictive biomarkers for pembrolizumab efficacy is of critical importance, pharmacodynamics measurements and search for biological/immunological correlates are planned on pre- and post-treatment tissue and blood samples regularly collected before, during, and after treatment.

### Statistics

The PELICAN trial is designed and powered to demonstrate that experimental group (arm B) achieves a pCR rate higher than a predefined undesirable rate of 20%. To reject the null hypothesis of a truly inefficient regimen (H0: p≤20%) at 5% error risk, following a Simon’s 2-stage optimal procedure, a total of 54 patients in arm B is necessary to obtain a power of 90% assuming a true pCR rate of 40%. Furthermore, to reach a power of 80% to reject the null hypothesis at 5% error risk in HR-negative (arm B), there must be at least 32 HR-negative patients and no more than 25 HR-positive patients to be recruited in arm B. A total of 27 patients are planned to be enrolled in the control arm (arm A), leading to a total of 81 patients planned. Predefined pCR of 20% was set-up according to several studies demonstrating a pCR rate between 20% and 28% in HER2-negative IBC receiving anthracyclines-taxanes NACT. The duration of enrolment is planned to 24 months. The randomized phase II design was selected to provide a control arm not directly compared to the experimental arm but allowing verifying the expected pCR rate with a conventional NACT in the selected population.

According to a 2-stage optimal design, an interim analysis is planned when 19 patients will be evaluable for the primary endpoint (pCR) in the experimental arm. At this interim evaluation, the study will stop for futility if no more than 4 patients have a documented pCR. If the patient accrual continues, the true pCR rate following NACT plus pembrolizumab will be estimated at the end of study with 90% confidence interval in all patients who received at least one dose of pembrolizumab. The overall hypothesis of a truly ineffective experimental arm will be accepted if the lower bound of the above estimated 90% bilateral confidence interval (CI) is inferior to 20%, or equivalently if no more than 15 patients out of a total of 54 evaluable patients have a documented pCR. In addition, the true pCR rate in patients enrolled in the HR-negative (TN IBC) strata will also be estimated. In this subgroup analysis, the true pCR rate will be estimated with 90% bilateral CI using an exact method for binomial proportions in one-stage clinical trials. The hypothesis of a truly ineffective experimental arm in this subgroup of interest will be accepted if the lower bound of the above estimated 90% exact bilateral CI is inferior to 20% and rejected otherwise.

According to standard practice in phase I studies, run-in phase conducted will enroll a maximum of 6 patients who completed 21 days after the first administration in two consecutive sub-cohorts (3+3). If at least 2 out of the 3 patients enrolled in the first sub-cohort report a DLT episode, the accrual of patients will be stopped, and the combination will be declared too toxic to warrant further investigation. If 1 or less than 1 patient (≤1 patient) reported a DLT in the first sub-cohort, 3 additional patients will be enrolled in the run-in phase. At the end, the combination will be declared sufficiently safe if less than 2 patients report a DLT out of the 6 evaluable patients enrolled in the run-in phase.

The incidence of reported adverse events during the treatment period will be summarized according to the treatment arm, by primary system organ class, CTCAE v5.0 severity grade, type of adverse event, and relationship to the study drug. Locally assessed pCR rate will be estimated with 90% exact confidence intervals. Time-to-event outcomes will be censored at the time of last follow-up visit. IDFS, EFS and OS will be estimated using the Kaplan-Meier method. Pointwise estimations for 3-year and 5-year IDFS, EFS, and OS will be provided with corresponding 90% asymptotic confidence interval.

## Conclusion

To our knowledge, the PELICAN-IPC 2015-016/Oncodistinct-003 study (NCT03515798) is the first one to investigate the efficacy of ICI in patients with IBC, a rare but difficult-to-treat form of BC. Pembrolizumab is combined to chemotherapy in the neoadjuvant setting. Even if the recent most promising results remain modest in BC compared with more immunogenic cancers such as lung cancer or melanoma, they are significant notably in metastatic TNBC when combined to chemotherapy. Furthermore, IBC display few molecular characteristics that may suggest higher efficiency than in non-IBC: more frequent TN subtype, more frequent PD-L1-positivity and higher TMB independently from the molecular subtypes. Enrolment began in July 2018 and the estimated study completion date is 2022.

## Data Availability Statement

The original contributions presented in the study are included in the article/supplementary material, further inquiries can be directed to the corresponding author.

## Ethics Statement

The studies involving human participants were reviewed and approved by Comité de Protection des Personnes IIe de France VII (hôpital de Bicêtre, Le Kremlin Bicêtre, FRANCE). The participants or legal guardian provided written informed consent to participate in this study.

## Author Contributions

AG was involved in the conceptualization of the manuscript. AB wrote the first draft of the manuscript. FB and AG revised the manuscript. AB was in charge of the tables and figure. All authors contributed to the article and approved the submitted version.

## Funding

This study was designed and led by the study investigators. MSD France provides financial support and the supply of the study drug for the trial.

## Conflict of Interest

AG reports travel expenses, accommodation, and meeting registration from Astra Zeneca, Pfizer, Roche, and Novartis. 

The remaining author declares that the research was conducted in the absence of any commercial or financial relationships that could be construed as a potential conflict of interest.
